# The OptimaMed intervention to reduce medication burden in nursing home residents with severe dementia: results from a pragmatic, controlled study

**DOI:** 10.1186/s12877-023-04222-4

**Published:** 2023-08-28

**Authors:** Edeltraut Kröger, Machelle Wilchesky, Michèle Morin, Pierre-Hugues Carmichael, Martine Marcotte, Lucie Misson, Jonathan Plante, Philippe Voyer, Pierre Durand

**Affiliations:** 1 Centre d’excellence sur le vieillissement de Québec, CIUSSSCN, Hôpital du Saint-Sacrement, 1050, Chemin Sainte-Foy, Québec (Québec), G1S 4L8 Canada; 2 Université Laval, Faculté de pharmacie, Pavillon Ferdinand Vandry, 1050 Avenue de La Médecine, Québec, Québec G1V 0A6 Canada; 3 Institut sur le vieillissement et la participation sociale des aînés, Université Laval, Hôpital du Saint-Sacrement, bureau L2-42, 1050, Chemin Sainte-Foy, Québec (Québec), G1S 4L8 Canada; 4 McGill University, Faculty of Medicine and Health Sciences, 3605, Chemin de La Montagne, Montreal (Québec), H3G 2M1 Canada; 5 Lady Davis Institute for Medical Research, Jewish General Hospital, 3755 Chem. de La Côte-Sainte-Catherine, Montréal, (Québec) H3T 1E2 Canada; 6 Donald Berman Maimonides Centre for Research in Aging, 5795 Av. Caldwell, Côte Saint-Luc, Montreal (Québec), H4W 1W3 Canada; 7 Université Laval, Faculté de médecine, Pavillon Ferdinand Vandry, 1050 Avenue de La Médecine, Québec (Québec), G1V 0A6 Canada; 8 Université Laval, Faculté des sciences infirmières, Pavillon Ferdinand Vandry, 1050 Avenue de La Médecine, Québec (Québec), G1V 0A6 Canada

**Keywords:** OptimaMed, Deprescribing, Inappropriate medication, Nursing home, Dementia

## Abstract

**Background:**

Nursing home (NH) residents with severe dementia use many medications, sometimes inappropriately within a comfort care approach. Medications should be regularly reviewed and eventually deprescribed. This pragmatic, controlled trial assessed the effect of an interprofessional knowledge exchange (KE) intervention to decrease medication load and the use of medications of questionable benefit among these residents.

**Methods:**

A 6-month intervention was performed in 4 NHs in the Quebec City area, while 3 NHs, with comparable admissions criteria, served as controls. Published lists of “mostly”, “sometimes” or “exceptionally” appropriate medications, tailored for NH residents with severe dementia, were used. The intervention included 1) information for participants’ families about medication use in severe dementia; 2) a 90-min KE session for NH nurses, pharmacists, and physicians; 3) medication reviews by NH pharmacists using the lists; 4) discussions on recommended changes with nurses and physicians. Participants’ levels of agitation and pain were evaluated using validated scales at baseline and the end of follow-up.

**Results:**

Seven (7) NHs and 123 participants were included for study. The mean number of regular medications per participant decreased from 7.1 to 6.6 in the intervention, and from 7.7 to 5.9 in the control NHs (*p*-value for the difference in differences test: < 0.05). Levels of agitation decreased by 8.3% in the intervention, and by 1.4% in the control NHs (*p* = 0.026); pain levels decreased by 12.6% in the intervention and increased by 7% in the control NHs (*p* = 0.049). Proportions of participants receiving regular medications deemed only exceptionally appropriate decreased from 19 to 17% (*p* = 0.43) in the intervention and from 28 to 21% (*p* = 0.007) in the control NHs (*p* = 0.22). The mean numbers of regular daily antipsychotics per participant fell from 0.64 to 0.58 in the intervention and from 0.39 to 0.30 in the control NHs (*p* = 0.27).

**Conclusions:**

This interprofessional intervention to reduce inappropriate medication use in NH residents with severe dementia decreased medication load in both intervention and control NHs, without important concomitant increase in agitation, but mixed effects on pain levels. Practice changes and heterogeneity within these 7 NHs, and a ceiling effect in medication optimization likely interfered with the intervention.

**Trial registration:**

The study is registered at ClinicalTrials.gov: # NCT05155748 (first registration 03–10-2017).

**Supplementary Information:**

The online version contains supplementary material available at 10.1186/s12877-023-04222-4.

## Background

Worldwide, the prevalence of dementia among older adults is high, and the prevalence of persons living with severe dementia is on the rise [1–4]. With accelerated population aging [5], the number of Canadians living with dementia is also increasing: the Public Health Agency of Canada (PHAC) estimated that in 2017 there were nearly 452 000 older adults, i.e. aged 65 years or above, living with dementia, or 6.7% of this age group [6]. A large proportion of these older adults may eventually need nursing home (NH) placement to compensate for the functional decline associated with neurocognitive disorders. As NH residents advance to the severe stage of dementia, they are close to the end of their lives und should benefit from a comfort care approach [7].

These residents also take a large number of medications: on average, in Canada in 2016, they were prescribed 9.9 medication classes, compared with 6.7 among seniors living in the community [8]. In the same year, nearly 70% of NH residents had at least one potentially inappropriate medication according to the Beers criteria, compared to 49.4% of seniors overall. About half to two-thirds of NH residents will experience an adverse medication reaction within a year [8].

Optimal medication use in NH residents living with severe dementia, i.e. the use of well-tolerated medications for evidence-based indications, is a challenge [9, 10]. First, residents with dementia may be unable to express the discomfort related to an adverse reaction [11], tolerability is frequently reduced in this population because of polypharmacy [12], and finally, age-related physiologic and illness-related [13] changes may altogether lead to altered pharmacokinetic and pharmacodynamical responses [14]. Also, in the face of limited life expectancy, these residents may not benefit from preventive medications [15]. Moreover, the under recognition of severe dementia as a terminal disease by health professionals [15–17] may favour the maintenance of some curative or purely preventive treatments that would be stopped in other persons with the same life expectancy. In addition, dementia may lead to the increased use of some medications considered less appropriate in older adults. The Collaborative Approach to Optimize Medication Use for Older People in Nursing Homes (COME-ON) study examined factors associated with the use of benzodiazepine receptor agonists and concluded that dementia was a factor associated with their use [18]. Furthermore, practical concerns related to medication use may have burdensome consequences, such as dysphagia [19] or required repeated tests [20, 21]. As such, frequent reviewing of the medication regimens of NH residents with severe dementia is required, to adjust for evolving medical conditions, to avoid overtreatment and adverse medication events, and ultimately to improve well-being [22].

Several recently published studies have focused on the deprescribing of medications for the general NH resident population. A systematic review of 41 randomized controlled trials showed a 59% reduction in potentially inappropriate medications (PIMs) and a 24% reduction in hospitalizations for this population, when deprescribing interventions were used [23]. Two studies that focused on the impact of a pharmacist-led intervention showed favorable findings: a significant reduction in PIMs was shown by Hashimoto et al. [24] and Balsom et al., found that residents in the intervention group took an average of 2.88 fewer medications than those in the control group after 6 months of implementation [25].

The Beers criteria [26] and STOPP/START [27] consensus offer guidance related to medication prescribing for the general older adult population. While a small body of research suggested guidance specifically targeted for patients with severe dementia [7, 28], there remained a knowledge gap regarding implementation, which led to the OptimaMed approach. OptimaMed began with a scoping review to identify medication appropriateness criteria and successful interventions aimed at reducing medication load in seniors with severe dementia, and a 15-member Delphi panel of clinical experts that provided consensus regarding lists of medications deemed “generally”, “occasionally” or “exceptionally” appropriate were created for the context of Canadian NH settings [29]. A pilot study [30] then tested these lists (Additional files 1 and 2, in French) and in a 4-months pre-post feasibility study in three Quebec NHs; the intervention proved feasible and resulted in a reduction in the mean number of medications from 9.43 to 8.41 per resident without any concomitant clinically significant change in agitation or pain measurements. A mixed methods literature review then identified barriers for older adults and their families regarding polypharmacy and deprescribing, the results of which led to improvements in the information leaflet provided to families of NH residents (Additional file 3, in French) invited to the present study [31]. The aims of this present study were to assess the effectiveness of providing NH staff with knowledge exchange (KE) sessions and tools for reviewing, adjusting or discontinuing medications for NH residents with severe dementia on: 1) Reducing overall medication burden; and 2) the number of “exceptionally appropriate” medications, in the hopes that this would translate in improved, or, at least, no deterioration in agitation or pain/discomfort.

## Methods

### Study design

We performed a pragmatic, controlled study, following TREND guidance [32, 33], in seven, not randomly selected, publicly financed NHs located in the greater Quebec City region between July 2017 and August 2018. The participating nursing homes were selected in collaboration with administrators from the greater Quebec City Health Network, avoiding NHs which had 1) participated in the pilot study [30], 2) participated in the PEPS study [34], or 3) underwent some form of transformation. A random selection of intervention and control NHs was not feasible because the collaboration of the NH administration was needed for study participation and could not be facilitated for these NHs.

### Setting

In Canada, publicly funded NHs are under the governance of the Ministry of Health of each province, meaning there are differences in their management and organization across the country. In the province of Quebec, NH clinical care is provided by general practitioners (GPs) and pharmacists, both on a part-time basis, and with 24/7 coverage by registered nurses. Also, consistent with their mission to care for people with complex needs, NH admission requires an important loss of autonomy as measured by the iso-SMAF score (usually above 11 on a scale of 1 to 14). The scores of this assessment algorithm provide a numerical autonomy profile classification based on the SMAF tool [35], which itself is based on the WHO’s classification of impairments, disabilities and handicaps [36]. It measures 29 functions of 5 domains: basic activities of daily living, instrumental activities of daily living, mobility, communication, and mental functions.

### Ethical considerations

Because of the severity of their dementia, written and informed consent was obtained from the family or legal representative of each resident included in the study. The study was approved by the Quebec City Integrated Health and Social Services University Network (CIUSSSCN) Ethics review board (Ethics Certificate: SSPL-2016–2017-14). All collected information was kept confidential to preserve the anonymity of participants throughout the study and beyond.

### Study sample

To be eligible, participants had to have resided in the participating NH for a minimum of 2 months at the time of study inclusion, be 60 years of age or older, have a diagnosis of dementia of any etiology in their medical chart, graded as being severe as defined by the Reisberg scale [37], have an iso-SMAF score of 13 or 14, which is indicative of severe functional impairment, and to not be in end-of-life palliative care at the time of inclusion. No other exclusion criteria were applied. Letters of invitation to participate, and an information leaflet (Additional file 3, in French), detailing facts about medication use in this group, as informed by our scoping review on patients and their families, were sent to eligible residents’ families or legal guardians [31]. Participants in control NHs consented in the same fashion and received usual care, with care teams also including a pharmacist, a physician and registered nurses. It is of note that pharmacists, physicians, and nurses working in the control NHs were all employed by the same larger Quebec City Health Network as the staff in the intervention NHs.

### Intervention

This study implemented an enhanced OptimaMed approach as compared to the pilot study [30], which was primarily aimed at interprofessional teams and patients’ families. The first enhancement concerned the 90-min knowledge exchange (KE) session which presented the medication review guidance (MRG) tool , which was based on the published medication appropriateness list [29] used in the pilot study [30], and to which an algorithm was added to taper antipsychotics. The tapering algorithm is available at: https://deprescribing.org/wp-content/uploads/2018/08/AP-deprescribing-algorithm-2018-English.pdf).

The documents for the MRG tool (Additional file 1), developed in French, specifically for the NHs of the health network, can also be found at:

https://www.ciusss-capitalenationale.gouv.qc.ca/sites/d8/files/docs/ProfSante/MissionUniversitaire/CEVQ/cevq_medication_demence_severe_oct2015.pdf

and there is a list of specific medications used in NHs in the healthcare network at:

https://www.ciusss-capitalenationale.gouv.qc.ca/sites/d8/files/docs/ProfSante/MissionUniversitaire/CEVQ/cevq_medication_en_demence_severe_index.pdf

The second enhancement concerned the auditory for the KE session, that took place within 6 weeks after the study start date at each intervention NH. It now also addressed auxiliary nurses and orderlies, in addition to GPs, pharmacists and nurses who were present in the pilot study, given that these staff can be influential in the decision to initiate, increase or maintain certain medications, such as antipsychotics. Staff were invited to the training session by the NH management during their regular work shift, but participation remained voluntary. The KE session was animated by the research team, which presented a short introduction on study background, followed by a case vignette, presenting the MRG tool, the medication list, the tapering algorithm, and other published tools. This part was presented by a geriatrician (M Morin) with extensive experience in Continuous Education. During and after the case vignette, questions, answers, and comments were encouraged and an exchange took place. The session informed staff of the study rationale, the complexity of prescribing for seniors with severe dementia (e.g. metabolic changes, polypharmacy, frailty, etc.) and provided strategies aimed at optimizing specific medication regimens, such as less stringent targets for blood pressure or glycaemia [38, 39], alternatives to benzodiazepines as sleeping aides [40], and specific strategies for medication discontinuation (e.g. statins) [41]. Categories of appropriateness were discussed, and possible medication changes relating to a fictional case were presented by the geriatrician. Emphasis was placed on deprescribing antipsychotics [42–44] and additional coaching sessions to support changes [45] were provided by the study nurse, who was experienced in the implementation of practice change in NHs (L Misson). She also presented the study to a regular meeting of the residents’ committee of each intervention NH and participated in informal exchanges on medication appropriateness with the NH nurses and orderlies.

A primary medication regimen review was conducted shortly after the study start date by each NH pharmacist for each participating resident in the intervention NHs. Other reviews could be performed, on an as-needed basis, following changes in the resident’s clinical status. The pharmacotherapy-related suggested modifications were discussed with the resident’s nurse and GP. The decision to adjust medications was ultimately left to the clinical judgement of each GP, be it to increase doses or to deprescribe (i.e., tapering or stopping), with no interference from the research team. Meetings with family members were also conducted, in person, or as over the telephone meetings, for both intervention and control group participants, mostly related to the family care-giver’s consent for his/her family members’s participation. These meetings allowed to explore their thoughts about the appropriate use of medications.

### Measurements

Medication regimen information was collected from electronic pharmacy records specific to each NH, for each included resident, at study beginning and at the end of the six-months follow-up period. All active prescriptions at those points in time (i.e. those prescribed both regularly and *pro re nata* (PRN)) were included. For patients who died during follow-up, follow-up data on prescription medication use were collected at two weeks before death. This period was chosen as in prior research [30, 34], because it corresponds to the end-of-life care period in which specific care protocols apply in the included NHs, so that medication use is governed by purely end-of life care objectives, for which the OptimaMed criteria were not validated. Thus, medication changes happening during the end-of life care period had to be excluded from analyses. All prescriptions were categorized as per the Optimamed MRG tool.

The study nurse was responsible for all measurements in included resident participants. Pain and agitation were measured in all participants, twice for the aim of this study, at study beginning and at the end of follow-up. Pain and discomfort was assessed using the French version of the Pain Assessment Checklist for Seniors with Limited Ability to Communicate (PACSLAC-F) [46, 47], which is also routinely used in Quebec’s NHs and based on observations of the psychomotor activity or change of habits/behavior by the resident, and results in a score between 0 and 60. Agitation was assessed using the Cohen-Mansfield Agitation Inventory (CMAI), a 29-item scale with each item being rated for a frequency from never (“0)” to several times per hour (“7”), for a scale from 29 to 203, with a mean above “45” being considered as serious [48]. Also, to increase the safety monitoring of patients, the study nurse had an active role seeking information from the care team on whether included residents manifested adverse effects. Data pertaining to age, sex, and medical comorbidities were collected by the study nurse at study beginning from residents’ medical records.

### Data analyses

Descriptive statistics were computed for all variables collected at study beginning and for all resident outcomes for both study beginning and end of follow-up. The main comparison was the difference in medication regimens between the intervention and control groups at these points in time. Mean numbers of medications were modeled using repeated measures mixed Poisson regression accounting for intra-subject correlated residual errors and random NH effects. Models included group, time, and group by time interaction effects. Difference in differences tests were computed to compare the difference of results between study beginning (at 0 month) and the last date of assessment (at 6 months) between exposed and control groups. Analyses used an intention-to-treat approach [49], meaning that all participants in the intervention sites were analysed as having benefitted from the OptimaMed medication review as recommended by the KE activities, and all participants in the control sites were analysed as not having received this specific review. Such an approach has been priorly used in educational intervention studies [50].

The proportion of patients exposed to each of the three OptimaMed appropriateness of use categories were analyzed independently using repeated measure mixed logistic regression. Again, the statistical model included group, time, and group by time interaction effects as well as intra-subject correlated residual errors and random NH effects. CMAI and PACSLAC scores were modelled using linear repeated measures mixed regression models with group, time, and group by time interaction effects as well as intra-subject correlation residual errors and random NH effects. There were no data missing for medications, as electronic pharmaceutical records contain complete data of residents’ medications, including daily dosages. As per current practice in these NHs, all regular prescribed medications are reliably given to the residents. Cases of forgotten or lost medications must be reported as incidents. There were also little missing clinical data, therefore no imputation method was deemed necessary for our analyses. As a sensitivity analysis, all models were also adjusted for age, Charlson comorbidity index and time since NH admission. A significance level alpha of 0.05 was used. Data analyses were performed using SAS® software (SAS Institute, version 9.4, Cary, NC).

## Results

**Fig. 1:**
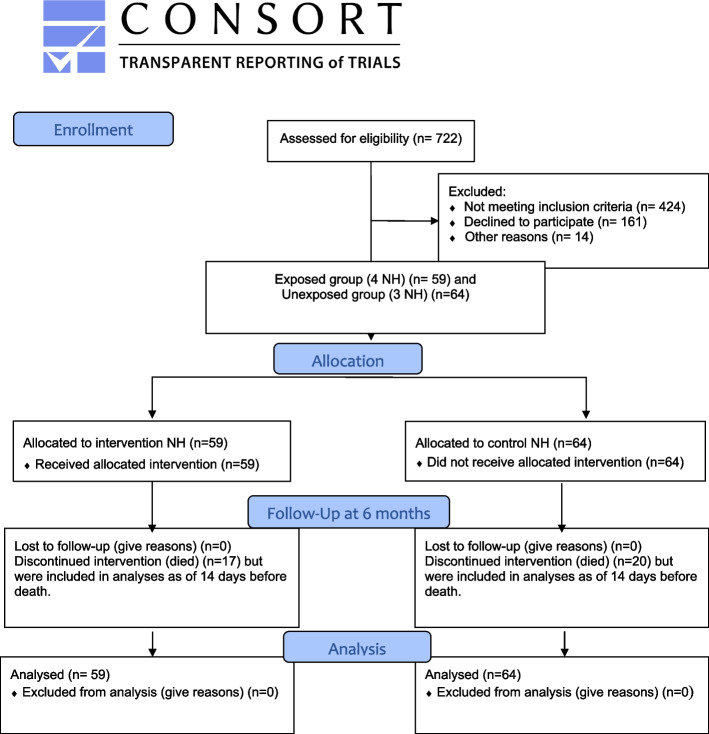
CONSORT 2010 Flow Diagram

The study included four intervention and three control NHs. Globally, 28 NH health professionals participated in the baseline KE sessions that took place during day shift: 6 GPs, 5 clinical pharmacists, 6 heads of NH care units (RNs or administrators), and 11 staff nurses (RNs or auxiliary nurses). A total of 722 residents were assessed for eligibility and 298 met the inclusion criteria. Among these, 161 (54%) declined, meaning that the family caregiver or guardian did not return a signed consent form. In many telephone-conversations between family members and the study nurse regarding study consent, family caregivers expressed hopelessness about their family member’s situation and could not imagine study participation could help significantly to improve this situation. Another 14 residents could not be included for other reasons, leaving 123 participants, of whom 59 lived in the intervention NH and 64 in the control sites. For the resident recruitment, please also refer to the flow-chart (Fig. 1, Consort Flow Diagram). In total, 37 residents representing 30% of the sample died during follow-up. All participants were included in final analyses on medication use, as explained above, but only surviving residents participated in the follow-up assessment of agitation and pain levels. The mean duration of follow-up for all participants, including the deceased, was 166 days or nearly 24 weeks in the intervention, and 188 days or nearly 27 weeks in the control group.

Baseline characteristics of participating residents are presented in Table 1. Mean age, percentage of women and Charlson comorbidity scores of participating residents were 83.2 vs. 86.6 years, 72.9% vs 81.3% women, and 6.88 vs 7.31 comparing intervention to controls, respectively. Overall, the intervention group NHs had a lower proportion of women, participating residents were a mean of 3.4 years younger, had a 5.9% lower Charlson comorbidity score, a higher mean score for discomfort and on the Cohen-Mansfield agitation inventory.

**Table 1:** Descriptive baseline characteristics, *n* = 123 participating residents

		Mean or % (± SD and/or range)	
		Intervention group (*n* = 59)	Control group (*n* = 64)
Age (years, mean) (range)		83.2 ± 9.5 (59.8 – 102.7)	86.6 ± 8.3 (70.1 – 105.4)
Female (%)		72.9	81.3
Follow-up (days, mean)		165.9	188.4
isoSMAF score (%)	10	3.39	1.56
	11	1.69	3.13
	12	11.86	10.94
	13	33.90	39.06
	14	49.15	45.31
Charlson Comorbidity Score (mean) (range)		6.88 ± 1.69 (3 – 10)	7.31 ± 1.83 (4 – 14)
CMAI (mean) (range)		39.1 ± 9.6 (30 – 80)	36.0 ± 8.1 (30 – 62)
PACSLAC-F short version (mean) (range)		4.93 ± 3.15 (1 – 17)	4.05 ± 3.01 (1 – 15)
Died during follow-up (n (%))		17 (29%)	20 (31%)

*SD* Standard deviation

Note: Total number of problematic behaviors observed during medication administration n at baseline: 5 in the control and 4 in the intervention NHs

Intervention outcomes are presented in Table 2.

**Table 2:** Modelled intervention outcomes^a^

	Intervention NHs (*n* = 59)			Control NHs (*n* = 64)			
Medication use	Study beginning	End of follow-up	*p*-vvalue**	Study beginning	End of follow-up	*p*-vvalue**	*p*-vvalue***
Total number of regular medications (n)	416	389		492	369		
Number of all medications per participant (mean ± SE)	8.15 ± 0.76	7.76 ± 0.73	0.28	10.73 ± 1.04	9.17 ± 0.91	**0.0001**	0.0806
Number of regular medications per participant (mean ± SE)	7.06 ± 0.45	6.60 ± 0.43	0.2126	7.69 ± 0.45	5.90 ± 0.40	** < 0.0001**	**0.0092**
Total number of regular “generally appropriate” medications (n)	140	127		156	121		
Total number of regular “sometimes appropriate” medications (n)	284	265		313	241		
Total number of regular “exceptionally appropriate” medications (n)	13	13		24	19		
Proportion of participants using regular “generally appropriate” medications (%)	86	81	0.4438	88	76	0.4583	0.9464
Proportion of participants using regular “sometimes appropriate” medications (%)	98	100	0.0917	98	98	0.1201	0.2770
Proportion of participants using regular “exceptionally appropriate” medications (%)	19	17	0.4331	28	21	**0.0069**	0.2168
Total number of residents with regular antipsychotics (n)^a^	38	34		25	19		
Number of regular antipsychotics per participant (mean ± SE)^a^	0.64 ± 0.09	0.58 ± 0.09	0.2141	0.39 ± 0.07	0.30 ± 0.06	**0.0199**	0.2687
CMAI (mean ± SE)	38.8 ± 1.4	35.6 ± 1.5	**0.0002**	36.0 ± 1.5	35.5 ± 1.5	0.5267	**0.0262**
PACSLAC-F short version (mean ± SE, range)	4.91 ± 0.41	4.29 ± 0.44	**0.0577**	4.06 ± 0.40	4.34 ± 0.44	0.3783	**0.0487**

*SD* Standard deviation

^a^Repeated measures, adjusted for random NH effect (difference in difference test)

^**^p-values for pre-post comparison

^***^p-values for change in time group comparison

Regarding objective 1, medication regimens of participating residents, in both control and intervention sites, included 171 different medications (ATC codes) at the beginning of the study and 149 such codes at the end of it. Mean numbers of regular daily medications fell from 7.69 to 5.90 in the control NHs (*p* < 0.05) and from 7.1 to 6.6 (*p* = 0.21) in the intervention NHs (Table 2).

At study beginning, the proportions of residents in the intervention and control sites, respectively, who received at least one regular medication considered “generally” appropriate, according to the MRG tool, were 86% and 81%; they were 98% and 98% for those considered “sometimes” and 19% and 28% for those considered “exceptionally” appropriate. While the proportions of residents receiving the “sometimes” appropriate medications stayed the same during the study period, the proportion of those receiving “generally” appropriate medications decreased from 86 to 81% in the intervention and from 88 to 76% in the control sites (all differences had a *p*-value > 0.05).

Regarding objective 2, the proportion with “exceptionally” appropriate medications decreased from 19 to 17% (ns) and from 28 to 21% (*p* = 0.007) in the intervention and control sites, respectively.

Finally, concerning objective 3, mean pain levels increased slightly (from 4.06 to 4.34 on a scale from 0 to 60, or by 6.9%, *p* = 0.3783) among residents in the control sites, but decreased a bit among the intervention site residents (from 4.9 to 4.3 or by 12.6%, *p* = 0.058) (Table 2). We assessed the use of opioids in both the control and intervention sites, at study beginning and at the end of follow-up. The mean number of regular opioid prescriptions per resident was 0.78 at study beginning and 0.41 at the end of follow-up, in the control sites, while these numbers were 0.78 and 0.80 at study beginning and the end of follow-up, respectively, in the intervention sites. The decrease in mean opioids in the control sites was significant (*p* < 0.001), as well as the difference in the differences between the control and intervention sites (*p* < 0.001). Overall, mean pain levels were below those observed in the pilot study and not clinically alarming; pain levels may have changed differentially among residents in the 7 study sites during the study period, explaining the differences in opioid use. Mean levels of agitation stayed similar in the control groups but decreased by 3.2 points (on a scale from 29 to 203) in the intervention group (*p* = 0.0002).

Regarding the use of antipsychotics as regular prescriptions (Table 2), there were some differences between control and intervention sites, with residents in the intervention NHs receiving roughly twice the mean daily number of regular antipsychotics (0.64) as compared to residents in the control NHs (0.39), with a significant (*p* = 0.02) decrease at the end of follow-up to 0.30 in the control sites, but without significant change (mean of 0.58) in the intervention site (*p* = 0.27 for the difference in differences). Very few problems with medication taking were observed (5 in 63 participants in the control NH and 4 in 59 participants in the intervention NH) as part of the assessments at study beginning [51]. However, procedures to administer crushed tablets mixed with yogurt or fruit purees had already been implemented for residents with dysphagia or severe dementia, at both the intervention and the control sites. The performed sensitivity analyses did not change any of these conclusions.

## Discussion

This pragmatic, controlled trial focussed on improving the appropriateness of regular medication use among NH residents affected by severe dementia and aimed at showing whether knowledge exchange based on a continuous education session and specific medication revision guidance would contribute to such an improvement. Optimamed was derived from a pilot pre-post feasibility study that included 44 residents, where a similar intervention produced a 12% reduction in the mean number of regular medications per resident in 2014, in NHs of the same Health Network in greater Quebec City, Canada [30]. The results of the present study show that during follow-up, the mean number of regular medications declined by 23.3%, from 7.7 to 5.9 in the control and by 6.5%, from 7.1 to 6.6, in the intervention site. They also show that both overall medication load and the proportions of residents receiving medications only “exceptionally appropriate” were reduced during the 6-months follow-up, among the intervention residents, but as much or more in the control sites. While this shows that the reduction of medication load in NH residents affected by severe dementia is possible, without a noticeable increase in discomfort or agitation, several limitations of the study may explain the greater reductions in the control sites.

First, the NHs that participated in both the pilot and the present study are, since April 2015, part of a large, publicly funded Quebec Integrated Health and Social Services University Network covering health and social services for mental health, rehabilitation, and geriatric services, both ambulatory and institutional, for a population of over 755 000 citizens within a geographic territory of 18 643 km^2^ (https://www.ciusss-capitalenationale.gouv.qc.ca/en/portrait), or nearly half the size of Switzerland, that includes 29 NHs. Given the recent creation of this vast healthcare network at the time of study beginning, and the fact that the Centre of Excellence in Aging in Quebec, which greatly facilitated the study, was part of this network, close administrative cooperation was essential for the intervention study to take place. NH managers needed to liberate staff to take part in the KE sessions during their shifts and the study necessitated observation and measurement of residents’ discomfort or agitation by a study nurse in all participating NHs, which are both sensitive topics for NH administrators and managers. Thus, when the authors presented the study to the network’s administration, certain NHs were designated for participation, in accordance with criteria set by the study team and listed above, but randomization of NHs could not be facilitated. Moreover, the NHs of the network are distributed over the vast territory of the network and the financial resources for this study limited our ability to deploy study staff to NHs far away from the research centre.

This health board’s department of pharmacy employs 91 pharmacists of whom each may provide (part time) clinical pharmacy services in several NHs, including some forms of staff rotation. In other words, practice improvement measures experienced in a specific facility may affect practice in a different facility through contact between pharmacists and changing deployment schedules. This holds, to a lesser extent, for nurses and physicians employed by the health network and working in NHs.

Second, in the time between the pilot and the present study, medication optimization in NHs became a priority in several countries, including Canada. In Quebec, two important initiatives took place between 2016 and 2019, which were both financed and endorsed by the Ministry of Health and Social Services and aimed at 1) the use of inappropriate antipsychotics in NHs, [52] and 2) reducing polypharmacy [34]. While the first initiative was performed in several NHs all over the province of Quebec, the latter one took place in the same health network as the present study. Although care was taken that this practice improvement intervention took place in neither the control nor the intervention sites of the present study, pharmacists, and to a lesser extent, physicians and nurses employed by the health network, may have worked in all the NHs included in the present study, while the OptimaMed intervention took place. Also, province-wide professional Continuous Education activities, particularly for pharmacists, frequently focussed on polypharmacy, deprescribing and medication optimization in NHs at the time of these studies. For example, Canadian guidelines for the deprescribing of antipsychotics in dementia [53] were published and largely circulated in 2015, after the pilot study. Thus, the present study took place in a period of overall emphasis on deprescribing of inappropriate medication in NHs, which likely contributed to improved overall care, at least up to the Covid-19 pandemic [54, 55].

Third, only 56.7% (161 of 284) of eligible residents could be included in the study. This rather low proportion of eligible residents whose families gave informed consent for study participation is certainly of concern. Reasons expressed during the conversations with the study nurse were most prominently distress about the family member’s disease state and hopelessness regarding disease evolution. In addition, there was apprehension regarding possible changes in the medication regimen. There remain important challenges to better inform families of NH residents affected by severe dementia on medication use for these persons, and to include them in shared decision making. Our leaflet for families (Additional file 3, in French) tried to address this need.

Fourth, while all residents in Quebec NHs are very frail, since a minimal SMAF score of 11 is needed for admission, related to high frailty, and all study participants were affected by severe dementia, residents in the intervention group were a mean of 3.4 years younger. Still, within a follow-up of over six months in the control sites, and of nearly 24 weeks in the intervention sites, 17 out of 59 residents died in the intervention sites and 20 out of 64 in the control sites, for a total of 37, or 29% in the intervention and 31% in the control sites, illustrating both the high level of frailty and the limited life expectancy of these residents. Despite the observation of similar mortality, there may have been clinical differences among residents in the different intervention and control sites, as visible in the differences in other resident characteristics in the intervention NHs, i.e. a lower proportion of women, lower comorbidity and somewhat higher mean scores for discomfort and agitation in the intervention NHs. Such differences may have affected decisions on medication prescribing, on adjustment and discontinuation differentially. Only a much larger multi-site cluster randomized trial may be able to avoid such mean baseline differences between intervention and control sites.

Another specific point to note is that in one of the control sites, a quality improvement project regarding end-of-life care had been realized in 2014. This project included training for all care team members, including physicians and pharmacist, while one of the NH nurses acted as an agent of change and was trained by the study nurse (L Misson) of the present study [56]. It is possible that this prior study had motivated the staff team to optimise medication use among vulnerable residents nearing the end of their life within the present study, although they acted as a control site.

There may also exist a “ceiling effect”, meaning that it might be unlikely that mean numbers of regular medications for frail NH residents with advanced dementia, in the age group well above 80 years, can stay significantly and consistently below 6 or 7 regular daily medications. Thus, a French study from 2021 among 800 NH residents with a mean age of 86 years found a mean number of daily medications of 8.5 [57]. A 2019 study of 103 NH residents in Norway concluded on some recent improvements regarding medication appropriateness in NH, while the mean number of regular daily medications stayed at 7.2, for a population of NH residents comparable to the present study [58]. Also the PEPS study [34] which took place in the same health board as this study, but included all NH residents regardless of diagnosis at admission, arrived at a mean number of 6.9 regular medications by the end of follow-up.

Finally, we did assess the numbers of daily “as needed” medications in the participants’ files (but not the dispensed “as needed” doses, since this information is not on the computer files), which were and stayed lower in the intervention sites (daily mean of 8.2 medications if “as needed” ones are included, before, and of 7.8 after the intervention) than in the control sites (daily mean number of 10.7 before and 9.2 after the intervention). It is of note that during the process of deprescribing a phase of “as needed use” may facilitate this process for certain medications, by allowing to consider day-to-day variations in the residents’ health. The practice of including “as needed” medications in the NH resident’s files varies among NHs and depends largely on physician and nurse practices. Thus, if the “as needed” medications were given frequently in the control sites, the mean of 5.9 daily medications at the end of the follow-up might be an underestimation of the actual medication use. Unfortunately, it is very difficult to analyse which medications were given on a “as needed” basis in a multi-site study.

It is important to stress that medication revision for NH residents suffering from severe dementia and aimed at reducing less appropriate medications stems from the desire to provide comfort care in the presence of severe dementia [7, 59–61]. This approach is the main motivation for the present study; it is however not merely based on remaining life expectancy, but on the conviction that residents with severe dementia benefit more from increased comfort than from increased life expectancy [7]. In this context, medication appropriateness categories serve as a guidance and should never replace clinical judgement by the resident’s physician and care team. Instead care decisions should consider all clinical, psychological, and social characteristics of the resident and their family. These considerations are reflected by the term “exceptionally appropriate” medications, which was chosen during the Delphi panel approach which had served to validate these categories and in which members expressed the necessity to always make individualized therapeutic decisions [29].

Mean daily antipsychotics were well below 1 medication of this class per resident, in both intervention and control sites, at study beginning and the end of follow-up, but the intervention did not result in any decrease in the intervention site, whereas usage did decrease in the control NH of the same health board: among the 64 residents in the control NH there were 25 who had at least one regular prescription for antipsychotics, which are also part of the “sometimes appropriate” medications, and this number fell to 19 residents at the end of follow-up, while in the intervention NH these numbers were 38 out of 59 at the beginning and 34 at the end of follow-up. Against expectations, there was one control NH, where the number of residents who used at least one regular antipsychotic decreased from 11 residents (40%) among 27 who participated, to 3 residents (11%) at the end of follow-up. Although we were not able to include dispensed “as needed” doses and day-to-day dose variations, the intervention did not seem to have the intended overall impact on a decreased use of antipsychotic agents, despite their considerable risk of serious adverse effects [62–65].

Our intervention built upon earlier work by Garfinkel et al. who incorporated evidence for medication indication, effectiveness, and adverse effects, as well as patient circumstances and continuation preferences into their approach [66, 67]. In Quebec public NHs clinical pharmacists are part of the regular care team, physically present and increasingly involved in medication adjustment, revision and, in specific circumstances, prescribing [34]. In 2019, the systematic review and meta-analysis by Kua et al. analyzed data from 41 randomized controlled trials on the reduction of overall medication burden or potentially inappropriate medications among NH residents, and concluded that different deprescribing intervention strategies led to an overall 59% reduction in PIMs [23]. Nine of these 41 studies were about interventions led by pharmacists, alone or together with nurses, and six relied on medication review, while pharmacists were involved in 16 trials.

In a Dutch cluster randomized trial to discontinue inappropriate medications [68], physicians in collaboration with pharmacists performed one multidisciplinary, multistep medication review (3MR) for NH residents. After a mean follow-up of 144 days, at least one inappropriate medication was discontinued according to the STOPP criteria [69] for 39% of participants, as compared to 29.5% in the control group, for an adjusted relative risk of 1.23 (95% CI 1.02; 1.75), while there was no deterioration of clinical outcomes. A significant reduction in PIMs was shown by Hashimoto et al. [24] in a deprescribing-focused medication review by a pharmacist among 45 NH residents. In Newfoundland, Canada, 78 deprescribing recommendations were made, 85.1% were implemented and residents in the intervention group took an average of 2.9 fewer medications than those in the control group after 6 months of implementation, although in 14.9% of cases medications had to be restarted because of recurring symptoms [25].

## Conclusions

This pragmatic, controlled study tested the effects of an interdisciplinary intervention comprising KE and medication review guidance for NH residents with severe dementia. While overall reduction of medication load was observed in both the control and the intervention NH, general practice improvement strategies aimed at all pharmacists and physicians working in the greater health network, and possibly a ceiling effect on the reduction of mean daily medications, likely contributed to this result. However, the interactions with pharmacists, nurses, and physicians during the KE sessions in this study confirmed that there is an ongoing need for education of clinicians on the considerations regarding medication appropriateness among NH residents with severe dementia. Guidance specific to these NH residents may still contribute to improved medication use among this particularly vulnerable population.

To counter the limitations of this pragmatic controlled study, a large, multi-site, cluster randomized trial is planned to evaluate the effects of the OptimaMed-LTC intervention, i.e. a KE session on medication appropriateness, medication adjustment and deprescribing in NH residents with severe dementia, based on validated and updated criteria on appropriateness in these particularly vulnerable residents. Moreover, the expanded roles of pharmacists already observed in several countries, including Canada, increasingly allow pharmacists to adjust medication dosage, including reduction to zero. Pharmacists may also prescribe some medications, i.e. use restricted prescribing privileges. Future studies should assess the effects of NH pharmacists’ medication review, adjustment and deprescribing on residents’ quality of life, agitation, and discomfort, particularly among those who are very frail or have severe dementia.

### Supplementary Information

**Additional file 1 .**

**Additional file 2 .**

**Additional file 3 .**

## Data Availability

All study data are kept in a safe and confidential manner at the Centre de recherche du CHU de Québec, Université Laval, until 2025 or 7 years after the end of the study. Access to these data may be granted upon reasonable request to the corresponding author.

## Abbreviations

CI: Confidence Intervals
CE-KE: Continuous education knowledge exchange
CMAI: Cohen-Mansfield Agitation Inventory
FAMS: Functional Autonomy Measurement System
GP: General Practitioners
GLMM: Generalized Linear Mixed Models
HSSB: Health and Social Services Board
KE: Knowledge Exchange
3MR: Multistep Medication Review
NH: Nursing Homes
OR: Odds Ratio
PACSLAC-F-tool: Pain Assessment Checklist for Seniors with Limited Ability to Communicate
p: Probability
FAST: Functional Assessment Staging Test
SD: Standard Deviation
STOPP-START: Screening Tool Of Older People's Prescription- Screening Tool To Alert To Right Treatment
