# Supplementary material for: The OptimaMed intervention to reduce medication burden in nursing home residents with severe dementia: results from a pragmatic, controlled study
Source: BMC Geriatr. 2023 Aug 28;23:520. doi: 10.1186/s12877-023-04222-4 (PMC10464023; doi:10.1186/s12877-023-04222-4)
Supplement: Supplementary file 1 — Additional file 1 . [file 12877_2023_4222_MOESM1_ESM.pdf]

Médicaments pour les patients atteints de démence sévère en CHSLD: généralement, parfois, ou exceptionnellement appropriés

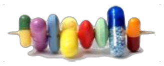

Fonds de la recherche en santé Québec

Réseau Québécois de Recherche sur le Vieillissement

Donald Berman Maimonides Medical Research Foundation / Fondation de recherche médicale

Fondation des hôpitaux Enfant-Jésus – Saint-Sacrement

CHU de Québec — Centre d'excellence sur le vieillissement de Québec

Dans le cadre de soins de qualité, l'utilisation judicieuse des médicaments contribue à minimiser les effets secondaires de ceux-ci et à optimiser le traitement.
Ce document a été réalisé par une équipe du Centre d'Excellence sur le Vieillissement de Québec pour faciliter l'optimisation du traitement pharmacologique (révision du profil pharmacologique et ajustements) des résidents de CHSLD âgés et atteints de démence sévère (stade 7 de l'échelle FAST).

Une revue de la littérature scientifique, notamment des travaux de Holmes et collègues,[1] a amené un panel d'experts cliniciens du Québec à s'entendre sur une catégorisation de médicaments (liste non exhaustive) en **généralement**, **parfois**, ou **exceptionnellement** appropriés en démence sévère.[2]
En découlent les tableaux suivants qui indiquent les classes de médicaments appartenant à chaque catégorie, leur code AHFS et des exemples de médicaments (*nom commercial entre parenthèses*). Un index des médicaments catégorisés est présenté en annexe.

Ni les agences subventionnaires, ni les auteurs ou leurs organisations affiliées n'ont d'intérêt en jeu dans les décisions prises par les professionnels utilisant ce document comme guide pour l'optimisation de la médication.

I : Médicaments généralement appropriés

Code AHFS – Classe médicamenteuse	Explications sommaires
12:12.08 - **Bronchodilatateurs inhalés**<br>Formotérol (*Oxeze*), Salbutamol (*Salvent, Ventolin*), Salmétérol (*Serevent*), Terbutaline (*Bricanyl*)	• Généralement appropriés, tant que l'usager est capable de **collaborer** à leur inhalation.
28:08.08 - **Analgésiques narcotiques**<br>Codéine, Fentanyl (*Duragésic*), Hydromorphone (*Dilaudid*), Morphine (*Eslon, Kadian, M.O.S., M-Slong, Statex*), Oxycodone (*Supeudol, Oxy-Contin, Oxy-IR*)	• La **douleur est prévalente** chez la personne âgée, même en présence de démence.<br>• Par contre, la mépéridine (*Demerol*) est un **mauvais choix** en gériatrie.
28:08.92 - **Analgésiques et antipyrétiques**<br>Acétaminophène (*Abenol, Tylenol, Novo-Gesic, Atasol, Tempra*)	• Comme **analgésique ou antipyrétique**.
28:12.92 - **Anticonvulsivants**<br>Carbamazépine (*Tégrétol*), Divalproex (*Epival*), Gabapentine (*Neurontin*), Lamotrigine (*Lamictal*), Lévétiracétam (*Keppra*), Prégabaline (*Lyrica*), Topiramate (*Topamax*), Acide valproïque (*Valproic, Depakene*), Vigabatrin (*Sabril*)	• Pour éviter l'inconfort des **convulsions**, d'autant que la démence (maladie d'Alzheimer, démence vasculaire) peut en abaisser le seuil.<br>• Évaluation au cas par cas pour la gabapentine et la prégabaline.
28:24.08 - **Benzodiazépines**<br>Lorazepam (*Ativan*), Oxazépam (*Sérax*), Temazépam (*Restoril*)	• En plus des approches non pharmacologiques, en cas d'anxiété ou d'insomnie non contrôlées. Non recommandées en cas d'agitation ou le delirium.<br>• Éviter les autres benzodiazépines.
52 - **Collyres lubrifiants**<br>(*Refresh, Gen Teal, Tears natural II, Systane*)	• Xérophtalmie fréquente et **inconfortable**.
84:04.08 - **Antifongiques topiques**<br>Ciclopirox olamine (*Loprox*), Clotrimazole (*Clotrimaderm, Canesten*), Kétoconazole (*Ketoderm*), Nystatine (*Nyaderm*)	• Pour éviter l'**inconfort** des mycoses cutanées ou buccales.

© Centre d'excellence sur le vieillissement de Québec 2015. Tous droits réservés.

II : Médicaments parfois appropriés (si la condition ou les besoins du résident le justifient)

Code AHFS - Classe médicamenteuse	Explications sommaires
4:08.00 - **Antihistaminiques** de 2e génération<br>Loratadine (*Claritin*)	• Pour **allergies**, démangeaisons, urticaire. Peuvent **exacerber** les symptômes de démence.
8:12 - **Antibactériens** (administration orale)<br>Toutes les classes, selon la stratégie thérapeutique	• Les antibiotiques peuvent être appropriés selon le niveau d'intervention thérapeutique, l'agent infectieux et le type d'infection.
8:18.28 - **Antiviraux** (inhibiteurs de la neuramididase, administration orale) : Oseltamivir (*Tamiflu*)	• Lors d'éclosions d'**influenza** si recommandation de la santé publique.
52:04.04 - **Anti-infectieux O.R.L.O.**	• Contribuent parfois au **confort**.
52:04.20 - **Antiviraux O.R.L.O.**<br>Trifluridine (*Viroptic*)	• Contribuent parfois au **confort** (herpès).
12:16.04 - **Bloquants α-adrénergiques urosélectifs**<br>Tamsulosine (*Flomax*), Silodosine (*Rapaflo*), Alfuzosine (*Xatral*)	• Pour éviter l'**inconfort** de la rétention urinaire.
20:12.1 - **Antiplaquettaires :** acide acétylsalicylique (*Aspirine*)	• N'est plus indiqué en prévention primaire.
24:12.08 - **Nitrates :** Nitroglycérine (*Nitro, Imdur, Minitran, Nitrodur, Nitropatch,* onguent *Nitrong*)	• En présence d'angine de poitrine chez un patient qui demeure **symptomatique**.
24:24.00 - **β-bloquants :** Acébutolol (*Rhotral, Sectral, Monitan*), Aténolol (*Tenormin*), Bisoprolol (*Monocor*), Carvédilol (*Coreg*), Labetolol (*Trandate*), Métoprolol (*Lopresor*), Sotalol (*Sotacor*)	• Pour **traiter les symptômes** de la maladie coronarienne, de certains troubles du rythme et de l'insuffisance cardiaque.
24:28 - **Bloquants du canal calcique**<br>*24:28.08* - **Dihydropyridines**<br>Amlopidine (*Norvasc*), Félodipine (*Plendil, Renidil*)<br>*24:28.92* - **Divers bloquants du canal calcique**<br>Diltiazem (*Tiazac, Cardizem*), Vérapamil (*Isoptin*)	• Pour soulager l'**inconfort** lié aux symptômes d'angine de poitrine.
24:32.04 - **Inhibiteurs enzyme conversion angiotensine**<br>Bénazépril (*Lotensin*), Cilazapril (*Inhibace*), Fosinopril (*Monopril*), Lisinopril (*Prinivil, Zestril*), Perindopril (*Coversyl*), Quinapril (*Accupril*), Ramipril (*Altace*), Trandolapril (*Mavik*)	• Viser des cibles de tension artérielle réalistes selon le niveau d'intervention thérapeutique et la condition du patient.
24:32.08 - **Antagonistes des récepteurs de l'angiotensine**<br>Candésartan (*Atacand*), Eprosartan (*Teveten*), Irbesartan (*Avapro*), Losartan (*Cozaar*), Olmésartan (*Olmetec*), Temisartan (*Micardis*), Valsartan (*Diovan*)	• Pour éviter l'**inconfort** de l'hypervolémie (décompensation cardiaque) en dysfonction ventriculaire gauche sévère.
28:16.04 - **Antidépresseurs** (autres que tricycliques)<br>Citalopram (*Celexa*), Fluvoxamine (*Luvox*), Paroxetine (Paxil), Sertraline (*Zoloft*), Venlafaxine (*Effexor*), Mirtazapine (*Remeron*)	• Dépression<br>• Parfois comme **co-analgésiques**.
28:16.08 - **Antipsychotiques**<br>Aripiprazole (*Abilify*), Clozapine (*Clozaril*), Halopéridol (*Haldol*), Olanzapine (*Zyprexa*), Quétiapine (*Seroquel*), Rispéridone* (*Risperdal*)*	• En plus des approches non pharmacologiques en **doses et durées minimales**, si détresse psychologique et SCPD.<br>• Éviter si delirium.
40:28 - **Diurétiques**<br>*40:28.08* - **Diurétiques de l'anse**<br>Furosémide (*Lasix*)<br>*40:28.16* - **Diurétiques épargneurs de potassium**<br>Amiloride (*Midamor*)<br>*40:28.20* - **Diurétiques thiazidiques**<br>Hydrochlorothiazide (*Hydrodiuril*)<br>*40:28.24* - **Diurétiques apparentés aux thiazidiques**<br>Chlorthalidone, Indapamide (*Lozide*), Metolazone (*Zaroxolyn*)	• Pour soulager les symptômes de l'insuffisance cardiaque tels que la **dyspnée**.<br><br>• Ne **pas** donner pour l'**œdème des membres inférieurs** attribuable à l'insuffisance veineuse.

* L'indication de la Rispéridone (*Risperdal*) relative à la démence a été restreinte à la prise en charge à court terme des symptômes d'agressivité ou des symptômes psychotiques chez les patients atteints de démence grave de type Alzheimer qui ne répondent pas aux approches non pharmacologiques, en présence d'un risque d'automutilation ou de blessure à autrui. L'indication n'inclut dorénavant plus le traitement des autres types de démence tels que les démences vasculaires ou mixtes. Santé Canada, février 2015.

Code AHFS - Classe médicamenteuse	Explications sommaires
52:40 - **Collyres anti-glaucome**<br>Brimonidine (*Alphagan*), Betaxolol (*Betoptic*), Lévobunolol, Timolol (*Timoptic*), Brinzolamide (*Azopt*), Dorzolamide (*Trusopt*), Carbachol, Pilocarpine, Bimatoprost (*Lumigan*), Latanoprost (*Xalatan*), Travoprost (*Travatan*)	• Pour éviter l'**inconfort** des crises glaucomateuses.
52:08.08 - **Corticostéroïdes O.R.L.O.**<br>Béclométhasone (*Rivanase*), Budésonide (*Pulmicort*, *Rhinocort*), Dexaméthasone (*Maxidex*), Fluorométholone (*FML, Flarex*), Fluticasone (*Flonase*), Mométasone (*Nasonex*), Prednisolone (*Pred*), Triamcinolone (*Nasacort*)	• Allergies O.R.L.O., rhinites allergiques **symptomatiques**.<br>• Tant que l'usager est capable de **collaborer** à leur inhalation.
56:08 - **Antidiarrhéiques**<br>Lopéramide (*Imodium*)	• Pour le confort lors de **diarrhée profuse** non infectieuse.
56:12 - **Laxatifs**<br>Toutes les classes, selon la stratégie thérapeutique	• Constipation rendant la personne **inconfortable**.
56:22.08 - **Antihistaminiques**<br>Dimenhydrinate (*Gravol*), Prochlorpérazine (*Stemetil*)	• Comme **antiémétique**, pour le confort, notamment si **vomissements**.
56:28.36 - **Inhibiteurs de la pompe à protons**<br>Dexlansoprazole (*Dexilant*), Esoméprazole (*Nexium*), Lansoprazole (*Prevacid*), Oméprazole (*Losec*), Pantoprazole (*Pantoloc*), Rabéprazole (*Pariet*)	• Quand facteurs de risque de **dyspepsie** ou **diathèse ulcéreuse**; en présence d'un RGO sévère ou d'un ulcère actif.
68:04 - **Corticostéroïdes** (administration orale)<br>Cortisone (*Cortone*), Dexaméthasone (*Decadron*), Hydrocortisone (*Cortef*), Méthylprednisolone (*Medrol*), Prednisone	• « **Flambée** » de maladies systémiques, telles que l'artérite temporale accompagnée de douleur; pour éviter l'insuffisance surrénalienne des utilisateurs chroniques.
68:20 - **Hypoglycémiants oraux**<br>*68:20.02* - **Inhibiteurs des alpha-glucosidases**<br>Acarbose (*Glucobay, Prandase*)<br>*68:20.04* – **Biguanides**<br>Metformine (*Glucophage*)<br>*68:20.20* - **Sulfonylurées**<br>Glyburide (*Diabeta*), Tolbutamide (*Mobénol*), Gliclazide (*Diamicron*)	• Lorsque le niveau d'intervention et la **qualité de vie** le justifient encore, pour éviter l'état hyperosmolaire.
68:20.08 - **Insulines**	• Lorsque le niveau d'intervention et la **qualité de vie** le justifient encore, pour éviter l'état hyperosmolaire.
68:36.04 - **Hormones thyroïdiennes**<br>Lévothyroxine (*Eltroxin, Synthroid*)	• Pour éviter l'**inconfort** des symptômes hypothyroïdiens (constipation, frilosité, aggravation du déclin cognitif, etc.)
68:36.08 - **Antithyroïdiens**<br>Méthimazole (*Tapazole*), Propylthiouracile (*PTU*)	• Pour éviter les conséquences de l'hyperthyroïdie telles que l'**agitation**.
84:04.12 - **Scabicides et pédiculicides**<br>Perméthrine (*Nix*) contre la gale ou les poux	• Pour des considérations de **santé publique**.
92:16.00 - **Antigoutteux**<br>Allopurinol (*Zyloprim*)	• Si goutte active et **symptomatique**.

III : Médicaments exceptionnellement appropriés (à éviter; très rarement justifiés)

Code AHFS - Classes de médicaments	Exemples d'exceptions
4:04.04 - **Antihistaminiques** 1e génération<br>Diphenhydramine (*Benadryl*), Hydroxyzine (*Atarax*)	• Pour réaction **allergique aiguë**, emploi de **courte durée**.
10:00.00 - **Antinéoplasiques**<br>Anastrozole (*Arimidex*), Bicalutamide (*Casodex*), Chlorambucil (*Leukeran*), Cyclophosphamide (*Procytox*), Cyprotérone* (*Androcur*)*, Flutamide (*Euflex*), Hydroxyurée (*Hydréa*), Nilutamide (*Anandron*)	• Pour usage palliatif : réduire le volume d'une **masse tumorale** causant de la douleur ou contribuer au soulagement des **métastases osseuses**.
12:08.08 - **Antimuscariniques/antispasmodiques**<br>Injection : Glycopyrrolate (*Robinul*), Scopolamine<br>Inhalateurs oraux : Ipratropium (*Atrovent*), Tiotropium (*Spiriva*), Glycopyrronium (*Seebri*)	• Pour diminuer les bruits respiratoires anormaux tels que ronchis.
20:12.04 - **Anticoagulants**<br>*20:12.04.08* - **Antagonistes de la vitamine K**<br>Warfarine (*Coumadin*), Nicoumalone (*Sintrom*)<br>*20:12.04.12* - **Inhibiteurs de la thrombine**<br>Dabigatran (*Pradaxa*)<br>*20:12.04.16* - **Héparines**<br>Héparine, Daltéparine (*Fragmin*), Enoxaparine (*Lovenox*), Fondaparinux (*Arixtra*), Nadroparine (*Fraxiparine*), Tinzaparine (*Innohep*)<br>*20:12.04.92* - **Inhibiteurs du facteur Xa**<br>Rivaroxaban (*Xarelto*), Apixaban (*Eliquis*)	Selon niveau d'intervention thérapeutique<br>• Porteurs de **valve cardiaque métallique**.<br>• Plus grand **risque de saignement** qu'avec la warfarine<br>• Pour **thrombophlébite profonde symptomatique**.
20:12.08 - **Anti-agrégants plaquettaires**<br>Clopidogrel (*Plavix*)	• Si **intolérance à l'aspirine**, selon le niveau d'intervention thérapeutique en accord avec l'usager et sa famille.
24:04.04 - **Antiarythmiques de classe I et III**<br>Amiodarone (*Cordarone*), Propafénone (*Rythmol*)	• Selon le **niveau d'intervention** thérapeutique en accord avec l'usager et sa famille.
24:04.08 - **Digoxine** (cardiotonique)<br>Digoxine (*Lanoxin*)	• Pour éviter les **palpitations** en FA rapide (justification excessivement rare).
24:06 - **Hypolipidémiants**<br>*24:06.04* - **Séquestrants de l'acide biliaire**<br>Cholestyramine (*Questran*), Colestipol (*Colestid*)<br>*24:06.06* - **Fibrates**<br>Bezafibrate (*Bezalip*), Fénofribrate (*Lipidil, Feno*), Gemfibrozil (*Lopid*)<br>*24:06.08* - **Inhibiteurs de l'HMG-COA réductase**<br>Atorvastatine (*Lipitor*), Fluvastatine (*Lescol*), Lovastatine (*Mevacor*), Pravastatine (*Pravachol*), Rosuvastatine (*Crestor*), Simvastatine (*Zocor*)<br>*24:06.92* - **Hypolipémiants divers**	• Selon le **niveau d'intervention** thérapeutique et sur insistance de la famille.
24:08.20 - **Vasodilatateurs à action directe**<br>Hydralazine (*Apresoline*)	• 4ième ligne en insuffisance cardiaque.
24:08.16 - **Agonistes α-adrénergiques**<br>Clonidine (*Catapres*)	• À éviter comme antihypertenseur.
28:08.04 - **Anti-inflammatoires non stéroïdiens (AINS)**<br>Célécoxib (*Celebrex*), Diclofénac (*Voltaren*), Diflunisal (Dolobid), Étodolac, Flurbiprofène (*Ansaid*), Ibuprofen (*Advil, Motrin*), Kétoprofène(*Orudis*), Méloxicam (*Mobicox*), Naproxène (*Naprosyn, Naprox*), Sulindac (*Clinoril*), Tenoxicam, Acide tiaprofénique (*Surgam*)	• Comme co-analgésiques, **seulement en fin de vie** quand on ne se préoccupe plus autant des effets secondaires nombreux.

* La cyprotérone (*Androcur*) est un anti-androgène qui a été placé avec les antinéoplasiques pour simplifier le tableau.

Code AHFS - Classes de médicaments	Exemples d'exceptions
28:16.04 - **Antidépresseurs tricycliques (TCA)**<br>Amitriptyline (*Elavil*)	• Seulement comme co-analgésiques en fin de vie, **pas comme antidépresseur**.
48:10.24 - **Antagonistes des récepteurs des leucotriènes**<br>Montélukast (*Singulair*), Zafirlukast (*Accolate*)	
68:08 - **Androgènes**<br>Danazol (*Cyclomen*), Testotérone (*AndroGel, Testim, Androderm, Andriol*)	• Peu d'indications valables.
68:16 - **Estrogènes et antiestrogènes**<br>*68:16.04* - **Estrogènes**<br>Estrogènes conjuguées (*Premarine*), Estradiol (*Estrace*)<br>*68:16.12* - **Antagonistes des estrogènes**<br>Clomifène (*Clomid*)	• Peu d'indications valables.
86:12.04 - **Spasmolytiques génito-urinaires**<br>Oxybutinine (*Ditropan*), Toltérodine (*Detrol*)	
92:16.00 - **Antigoutteux**<br>Colchicine	• **Crise de goutte**.
92:24.00 - **Bisphosphonates**<br>Alendronate (*Fosamax*), Alendronate/cholécalciférol (*Fosavance*), Risédronate (*Actonel*), Acide zolédronique (*Aclasta*)	• Une prise antérieure (> 5 ans) protège 1 à 2 ans après la cessation du traitement.
92:44 - **Immunosuppresseurs**<br>Azathioprine (*Imuran*), Cyclosporine (*Neoral*), Mycophénolate (*Myfortic*), Sirolius (*Rapamune*), Tacrolimus (*Prograf, Advagraf*)	• Peu d'indications valables.

Pour certains des médicaments qui leur ont été soumis, les experts cliniciens du Québec n'ont pu s'entendre sur une catégorisation (la catégorisation publiée par Holmes et collègues[1] est présentée à titre indicatif).

12: 04.00 **Inhibiteurs de l'acétylcholinestérase**<br>Donepezil (*Aricept*), Rivastigmine (*Exelon*), Galantamine (*Reminyl*)	Considérés jamais appropriés par Holmes et collègues[1]
28:92.00 - **Mémantine**<br>Mémantine (*Ebixa*)	Considérés jamais appropriés par Holmes et collègues[1]
28:08.04.24 - **Capsaïcine**<br>Capsaïcine	Considérée parfois appropriée par Holmes et collègues[1]
56:28.12 - **Antagonistes des récepteurs H_2 de l'histamine**<br>Cimétidine (*Tagamet*), Famotidine (*Pepcid*), Nizatidine (*Axid*), Raniditine (*Zantac*)	Considérés parfois appropriés par Holmes et collègues[1]
88 - **Vitamines**	Pas de consensus[1]

Références

1. Holmes HM, Sachs GA, Shega JW, Hougham GW, Cox Hayley D, Dale W. Integrating palliative medicine into the care of persons with advanced dementia: identifying appropriate medication use. J Am Geriatr Soc 2008;56:1306-1311.
2. Kröger E, Wilchesky M, Marcotte M, Voyer P, Morin M, Champoux N, Monette J, Aubin M, Durand PJ, Verreault R, Arcand M. Medication Use Among Nursing Home Residents With Severe Dementia: Identifying Categories of Appropriateness and Elements of a Successful Intervention. J Am Med Dir Assoc 2015;16:629 e1-17.

Remerciements

Le Centre d'excellence sur le vieillissement de Québec aimerait remercier les cliniciens qui ont participé au panel d'experts. Nos remerciements vont également à l'équipe du CEVQ qui a préparé ce document, plus particulièrement Edeltraut Kröger, professeure adjointe à la faculté de pharmacie de l'Université Laval, Michèle Morin, professeure de clinique à la faculté de médecine de l'Université Laval, Martine Marcotte, professionnelle de recherche, avec l'aide précieuse de Lise Grenier-Gosselin, pharmacienne, et du département de pharmacie du CSSS de la Vieille-Capitale.
