# Supplementary material for: The OptimaMed intervention to reduce medication burden in nursing home residents with severe dementia: results from a pragmatic, controlled study
Source: BMC Geriatr. 2023 Aug 28;23:520. doi: 10.1186/s12877-023-04222-4 (PMC10464023; doi:10.1186/s12877-023-04222-4)
Supplement: Supplementary file 2 — Additional file 2 . [file 12877_2023_4222_MOESM2_ESM.pdf]

Médicaments en démence sévère

Index des médicaments catégorisés

Généralement appropriés	Parfois appropriés	Exceptionnellement appropriés	PAS DE CONSENSUS

A	page
Abenol	*1*
Abilify	*2*
Acarbose	3
Accolate	*5*
Accupril	*2*
Acébutolol	2
Acétaminophène	1
acide acétylsalicylique	2
Acide tiaprofénique	4
Acide valproïque	1
Acide zolédronique	5
Aclasta	*5*
Actonel	*5*
Advagraf	*5*
Advil	*5*
**Agonistes α-adrénergiques**	**4**
Alendronate	5
Alendronate/cholécalciférol	5
Alfuzosine	2
Allopurinol	3
Alphagan	*2*
Altace	*2*
Amiloride	2
Amiodarone	4
Amitriptyline	5
Amlopidine	2
**Analgésiques narcotiques**	**1**
Anandron	*4*
Anastrozole	4
Andriol	*5*
Androcur (anti-androgène)	*4*
Androderm	*5*
AndroGel	*5*
**Androgènes**	**5**
Ansaid	*5*
**Antagonistes de vitamine K**	**4**
**Antagonistes des récepteurs de l'angiotensine**	**2**
**Antagonistes des récepteurs des leucotriènes**	**5**
ANTAGONISTES DES RÉCEPTEURS H_2 DE L'HISTAMINE	5
**Antiarythmiques** classe I & III	**4**
**Antibactériens oraux**	2
**Anticonvulsivants**	**1**
**Antidépresseurs** (non TCA)	**2**
**Antidépresseurs TCA**	**5**
**Antidiarrhéiques**	**3**
**Antifongiques** topiques	**1**
**Antihistaminiques** 1e génération	**4**
**Antihistaminiques** 2e génération	2
**Anti-infectieux O.R.L.O.**	**2**
**Anti-inflammatoires non stéroïdiens**	**4**
**Antinéoplasiques**	**4**
**Antipsychotiques**	**2**
**Antispasmodiques**	**4**
**Antithyroïdiens**	**3**
**Antiviraux O.R.L.O.**	**2**
Apixaban	4
Apresoline	*4*
ARICEPT	5
Arimidex	*4*
Aripiprazole	2
Arixtra	*4*
Aspirine	2
Atacand	*2*
Atarax	4
Atasol	*1*
Aténolol	2
Ativan	*1*
Atorvastatine	4
Atrovent	*4*
Avapro	*2*
AXID	5
Azathioprine	5
Azopt	*3*

B	
Béclocort (inhalateur)	3
Béclométhasone (ORLO)	3
Benadryl	*4*
Bénazépril	2
**Benzodiazépines**	**1**
**Bêta-bloquants**	**2**
Betaxolol	2
Betoptic	*2*
Bezafibrate	4
Bezalip	*4*
Bicalutamide	4
**Biguanides**	**3**
Bimatoprost	3
Bisoprolol	2
**Bisphosphonates**	**5**
**Bloquants du canal calcique**	**2**
**Bloquants alpha-adrénergiques urosélectifs**	**2**
Bricanyl	*1*
Brimonidine	3
Brinzolamide	3
**Bronchodilatateurs inhalés**	**1**
Budésonide (ORLO)	

C	
Candésartan	2
Canesten (topique)	1
CAPSAÏCINE	5
Carbachol	3
Carbamazépine	1
Cardizem	*2*
Carvédilol	2
Casodex	*4*
Catapres	*4*
Celebrex	*4*
Célécoxib	4
Celexa	*2*
Chlorambucil	4
Chlorthalidone	2
Cholestyramine	4
Ciclopirox olamine (topique)	1
Cilazapril	2
CIMÉTIDINE	5
Citalopram	2
Claritin	*2*
Clinoril	*4*
Clomid	5
Clomifène	5
Clonidine	4
Clopidogrel	4
Clotrimaderm (topique)	*1*
Clotrimazole (topique)	1
Clozapine	2
Clozaril	*2*
Codéine	1
Colchicine	4
Colestid	4
Colestipol	4
**Collyres anti-glaucome**	**3**
**Collyres lubrifiants**	**1**
Cordarone	*4*
Coreg	*2*
Cortef (oral)	3
**Corticostéroïdes**	**3**
Cortisone (oral)	3
Cortone (oral)	*3*
Coumadin	*4*
Coversyl	*2*
Cozaar	*2*
Crestor	*4*
Cyclomen	*5*
Cyclophosphamide	5
Cyclosporine	5
Cyprotérone*	4

D	
Dabigatran	4
Daltéparine	4
Danazol	5
Decadron (oral)	*3*
Depakene	*1*
Detrol	*5*
Dexaméthasone	3
Dexilant	*3*
Dexlansoprazole	3
Diabeta	*3*
Diamicron	*3*
Diclofénac	4
Diflunisal	4
Digoxine	4
Dilaudid	1
Diltiazem	2
Dimenhydrinate	3
Diovan	*2*
Diphenhydramine	4
Ditropan	*5*

**Diurétiques**	**2**
Divalproex	1
Dolobid	*4*
Donepezil	*5*
Dorzolamide	3
Duragésic	

E

Ebixa	*5*
Effexor	*2*
Elavil	5
Eliquis	4
Eltroxin	*3*
Enoxaparine	4
Epival	1
Eprosartan	2
Eslon	*1*
Esoméprazole	3
Estrace	5
Estradiol	5
Estrogènes conjuguées	5
Étodolac	4
Euflex	4
Exelon	*5*

F

Famotidine	*5*
Félodipine	2
Feno	*4*
Fénofribrate	4
Fentanyl	1
Flarex	*3*
Flomax	*3*
Flonase	*3*
Fluorométholone	3
Flurbiprofène	4
Flutamide	4
Fluticasone	3
Fluvastatine	4
Fluvoxamine	2
FML	*3*
Fondaparinux	4
Formotérol (inhalateur)	1
Fosamax	*5*
Fosavance	*5*
Fosinopril	2
Fragmin	*4*
Fraxiparine	*4*
Furosémide	2

G

Gabapentine (au cas par cas)	1
Galantamine	*5*
Gemfibrozil	4
Gen Teal	*1*
Gliclazide	3
Glucobay	*3*
Glucophage	3
Glyburide	3
Glycopyrrolate (injectable)	4
Glycopyrronium (inhalateur)	4
Gravol	*3*

H

Haldol	*2*
Halopéridol	2
Héparine	4
**Hormones thyroïdiennes**	**3**
Hydralazine	4
Hydréa	*4*
Hydrochlorothiazide	2
Hydrocortisone (oral)	3
Hydrodiuril	*2*
Hydromorphone	1
Hydroxyurée	4
Hydroxyzine	4
**Hypoglycémiants oraux**	**3**
**Hypolipémiants**	**4**

I

Ibuprofen	4
Imdur	*2*
**Immunosuppresseurs**	**5**
Imodium	3
Imuran	*5*
Indapamide	2
Inhibace	*2*
Inhibiteurs de l'acétylcholinestérase	*5*
**Inhibiteurs de l'HMG-COA réductase**	**4**
**Inhibiteurs de neuramididase** oraux	**2**
**Inhibiteurs de la pompe à protons**	**3**
**Inhibiteurs de la thrombine**	**4**
**Inhibiteurs des α-glucosidases**	**3**
**Inhibiteurs du facteur Xa**	**4**
**Inhibiteurs enzyme conversion angiotensine**	**2**
Innohep	*4*
**Insulines**	**3**
Ipratropium (inhalateur)	4
Irbesartan	2
Isoptin	*2*

K

Kadian	*1*
Keppra	*1*
Kétoconazole (topique)	1
Ketoderm	*1*
Kétoprofène	4

L

Labetolol	2
Lamictal	*1*
Lamotrigine	1
Lanoxin	*4*
Lansoprazole	3
Lasix	*2*
Latanoprost	3
**Laxatifs**	**3**
Lescol	*4*
Leukeran	*4*
Lévétiracétam	1
Lévobunolol	2
Lévothyroxine	3
Lipidil	*4*
Lipitor	*4*
Lisinopril	2
Lopéramide	3
Lopid	*4*
Lopresor	*2*
Loprox (topique)	*1*
Loratadine	2
Lorazepam	1
Losartan	2
Losec	*3*
Lotensin	*2*
Lovastatine	4
Lovenox	*4*
Lozide	*2*
Lumigan	*3*
Luvox	*2*
Lyrica (au cas par cas)	*1*

M

M.O.S	*1*
Mavik	*2*
Maxidex	*3*
Medrol (oral)	*3*
Méloxicam	4
Mémantine	*5*
Metformine	3
Méthimazole	3
Méthylprednisolone (oral)	3
Metolazone	2
Métoprolol	2
Mevacor	*4*
Micardis	*2*
Midamor	*2*
Minitran	*2*
Mirtazapine	2
Mobénol	3
Mobicox	*4*
Mométasone	3
Monitan	*2*
Monocor	*2*
Monopril	*2*
Montélukast	5
Morphine	1
Motrin	*4*
M-Slong	*1*
Mycophénolate	5
Myfortic	*5*

N

Nadroparine	4
Naprosyn	*4*
Naprox	*4*
Naproxène	4
**Narcotiques analgésiques**	**1**
Nasacort	*3*
Nasonex	*3*
Neoral	*5*
Neurontin (au cas par cas)	*1*
Nexium	*3*
Nicoumalone	4
Nilutamide	4
**Nitrates**	**2**
Nitro	*2*
Nitrodur	*2*
Nitroglycérine	2
Nitrong (onguent)	*2*
Nitropatch	*2*
Nix	*3*
Nizatidine	5
Norvasc	*2*
Novo-Gesic	*2*
Nyaderm	*1*
Nystatine (topique)	1

O

Olanzapine	2
Olmésartan	2
Olmetec	*2*

Oméprazole	3
Orudis	*4*
Oseltamivir	2
Oxazépam	1
Oxeze (inhalateur)	*1*
Oxybutinine	5
Oxycodone	1
Oxy-Contin	*1*
Oxy-IR	*1*

P

Pantoloc	*3*
Pantoprazole	3
Pariet	*3*
Paroxetine	3
Paxil	*2*
PEPCID	*5*
Perindopril	2
Perméthrine	3
Pilocarpine	3
Plavix	*4*
Plendil	*2*
Pradaxa	*4*
Prandase	*3*
Pravachol	*4*
Pravastatine	4
Pred	*3*
Prednisolone (ORLO)	3
Prednisone	3
Prégabaline (au cas par cas)	1
Premarine	*5*
Prevacid	*3*
Prinivil	*2*
Prochlorpérazine	3
Procytox	*4*
Prograf	*5*
Propafénone	4
Propylthiouracile	3
PTU	*3*
Pulmicort (inhalateur)	*3*

Q

Questran	*4*
Quétiapine	2
Quinapril	2

R

Rabéprazole	3
Ramipril	2
RANIDITINE	*5*
Rapaflo	*2*
Rapamune	*5*
Refresh	*1*
Remeron	*2*
REMINYL	*5*
Renidil	*2*
Restoril	*1*
Rhinocort	3
Rhotral	*2*
Risédronate	5
Risperdal	*2*
Rispéridone	2
Rivanase	*3*
Rivaroxaban	4
RIVASTIGMINE	*5*
Robinul (injectable)	*4*
Rosuvastatine	4
Rythmol	*4*

S

Sabril	*1*
Salbutamol (inhalateur)	1
Salmétérol (inhalateur)	1
Salvent (inhalateur)	*1*
**Scabicides et pédiculicides**	**3**
Scopolamine (injectable)	4
Sectral	*2*
Seebri (inhalateur)	*4*
Sérax	*1*
Serevent	1
Seroquel	*2*
Sertraline	2
Silodosine	2
Simvastatine	*4*
Singulair	*5*
Sintrom	*4*
Sirolius	5
Sotacor	2
Sotalol	2
**Spasmolytiques génito-urinaires**	**5**
Spiriva (inhalateur)	*4*
Statex	*1*
Stemetil	3
**Sulfonylurées**	**3**
Sulindac	4
Supeudol	*1*
Surgam	*4*
Synthroid	*3*
Systane	*1*

T

Tacrolimus	5
TAGAMET	*5*
Tamiflu	*2*
Tamsulosine	2
Tapazole	*3*
Tears natural II	*1*
Tégrétol	*1*
Temazépam	1
Temisartan	2
Tempra	*1*
Tenormin	*2*
Tenoxicam	4
Terbutaline (inhalateur)	1
Testim	*5*
Testotérone	5
Teveten	*2*
Tiazac	*2*
Timolol	2
Timoptic	*2*
Tinzaparine	4
Tiotropium (inhalateur)	4
Tolbutamide	3
Toltérodine	5
Topamax	*1*
Topiramate	1
Trandate	*2*
Trandolapril	2
Travatan	*3*
Travoprost	3
Triamcinolon	3
Trifluridine	2
Trusopt	*2*
Tylenol	*1*

V

Valproic	*1*
Valsartan	2
**Vasodilatateurs à action directe**	**4**
Venlafaxine	2
Ventolin	*1*
Vérapamil	2
Vigabatrin	1
Viroptic	*2*
VITAMINES	*5*
Voltaren	*4*

W

Warfarine	4

X

Xalatan	*3*
Xarelto	*4*
Xatral	*2*

Z

Zafirlukast	5
ZANTAC	*5*
Zaroxolyn	*2*
Zestril	*2*
Zocor	*4*
Zoloft	*2*
Zyloprim	3
Zyprexa	*2*
