# Supplementary material for: The OptimaMed intervention to reduce medication burden in nursing home residents with severe dementia: results from a pragmatic, controlled study
Source: BMC Geriatr. 2023 Aug 28;23:520. doi: 10.1186/s12877-023-04222-4 (PMC10464023; doi:10.1186/s12877-023-04222-4)

DES EXEMPLES?

Les médicaments visant à améliorer le confort et la qualité de vie du patient ont toujours leur place. Cependant, il est pertinent de questionner l'utilité de ceux qui sont donnés en prévention.

Par exemple :

- Devrait-on continuer les médicaments pour abaisser le cholestérol?
  - Il faut les prendre pendant au moins 3 ans pour qu'ils soient utiles à prévenir la maladie cardiaque;
  - Les douleurs musculaires sont un effet indésirable fréquent.
- Devrait-on continuer les médicaments contre la maladie d'Alzheimer même chez un patient dont la maladie est rendue à un stade avancé?
  - L'utilité est incertaine dans ce contexte;
  - Ces médicaments ont des effets indésirables tels que les nausées et la baisse d'appétit.

UNE PRESCRIPTION ADAPTÉE À LA SITUATION DE CHAQUE PERSONNE

- Il est recommandé de revoir la médication du patient si sa santé se détériore;
- Il faut considérer non seulement les avantages et les inconvénients de chaque médicament mais aussi la qualité de vie du malade et ce qui est dans son meilleur intérêt à ce stade de la maladie;
- En tant que personne ayant à cœur l'intérêt de votre proche, vous avez une place importante pour aider le médecin à décider ce qui est préférable pour lui ou elle.

VOUS AIMERIEZ EN PARLER?

L'équipe de recherche du projet « ***OptimaMed-LTC*** » peut vous fournir des informations supplémentaires.

Communiquez avec:

Lucie Misson, infirmière et coordonnatrice clinique du projet au **418 682-7511, poste 84839**.

Au besoin laissez un message, comprenant votre nom et votre numéro de téléphone, dans sa boîte vocale ou au numéro 418 682-7511, poste 82378.

LES MÉDICAMENTS CHEZ LA PERSONNE ÂGÉE EN SOINS DE LONGUE DURÉE

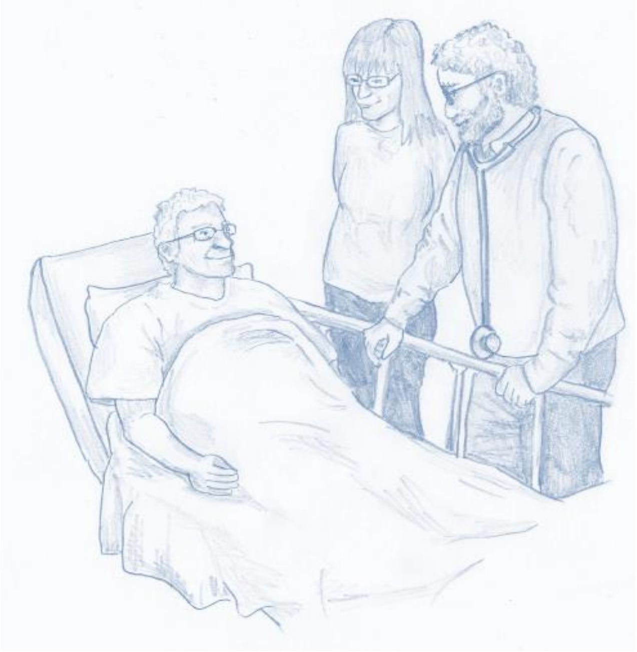

Adapté du document préparé par :

Centre de santé et de services sociaux –
Institut universitaire de gériatrie
de Sherbrooke

QUELQUES FAITS…

- Les personnes âgées admises en établissement de soins de longue durée souffrent la plupart du temps de plusieurs maladies (diabète, hypertension, maladie cardiaque, maladie d’Alzheimer, etc.).
- Elles doivent donc prendre plusieurs médicaments:
  - Actuellement, la moyenne est de 14 comprimés par jour
  - Certains patients prennent jusqu’à 30 comprimés par jour.
- Avec l’avance en âge, les reins et le foie sont habituellement plus lents à nettoyer le corps des résidus des médicaments ce qui augmente ainsi le risque d’effets secondaires indésirables.
- Plus une personne prend de médicaments, plus elle est à risque de présenter des effets indésirables.
- L’arrêt de certains médicaments qu’on croyait utiles jusqu’ici entraîne parfois, de façon surprenante, une amélioration de l’état du patient!

LA PROBLÉMATIQUE…

- L’utilité de certains médicaments n’est pas démontrée clairement. La plupart des études ont été faites sur des personnes plus jeunes et habituellement moins malades. Il n’est pas certain que les résultats de ces études s’appliquent aux personnes plus âgées dont la santé se détériore.
- L’administration de médicaments à des patients avec des troubles cognitifs comme la maladie d’Alzheimer crée des problèmes :
  - ces personnes ont souvent de la difficulté à avaler ce qui oblige les soignants à écraser les comprimés, et à les mélanger à la nourriture;
  - les difficultés de communication qui se développent à la longue peuvent empêcher les malades de rapporter les effets indésirables de leurs médicaments;
  - enfin plusieurs personnes refusent de prendre les médicaments parce qu’elles ne sont plus en mesure de comprendre leur utilité.

DES QUESTIONS SE POSENT

- Plusieurs médicaments prescrits ont pour but de prévenir des complications dans les années à venir.
- Chez les patients très âgés dont la santé s’est considérablement détériorée ou dont l’espérance de vie ne dépasse pas une ou deux années, les médecins se questionnent souvent sur la pertinence de continuer ces traitements préventifs.
- Le médecin ne veut pas priver le patient d’un traitement utile, mais ne veut pas non plus s’acharner à prescrire des médicaments peu utiles, ou même nuisibles.

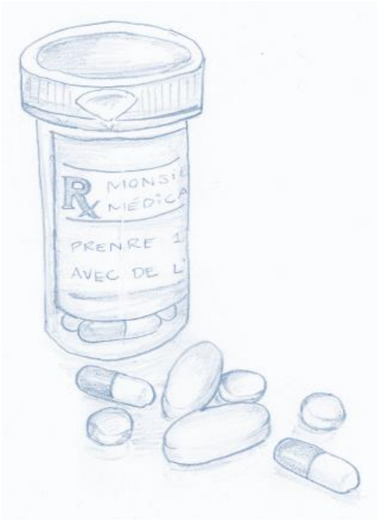

Supplement: Supplementary file 3 — Additional file 3 . [file 12877_2023_4222_MOESM3_ESM.pdf]
